# Indolediketopiperazine Alkaloids from *Eurotium cristatum* EN-220, an Endophytic Fungus Isolated from the Marine Alga *Sargassum thunbergii*

**DOI:** 10.3390/md15020024

**Published:** 2017-01-25

**Authors:** Feng-Yu Du, Xin Li, Xiao-Ming Li, Li-Wei Zhu, Bin-Gui Wang

**Affiliations:** 1 Laboratory of Marine Biology and Biotechnology, Qingdao National Laboratory for Marine Science and Technology, Key Laboratory of Experimental Marine Biology, Institute of Oceanology, Chinese Academy of Sciences, Nanhai Road 7, Qingdao 266071, China; fooddfy@126.com (F.-Y.D.); lixin871014@163.com (X.L.); lixmqd@aliyun.com (X.-M.L.); 2 College of Chemistry and Pharmacy, Qingdao Agricultural University, Changcheng Road 700, Qingdao 266109, China; qauliwei@163.com

**Keywords:** endophytic fungus, marine alga, *Eurotium cristatum*, indolediketopiperazine, bioactivity

## Abstract

Four new indolediketopiperazine derivatives (**1**–**4**), along with nine known congeners (**5**–**13**), were isolated and identified from the culture extract of *Eurotium cristatum* EN-220, an endophytic fungus obtained from the marine alga *Sargassum thunbergii*. The structures of thesecompounds were elucidated on the basis of extensive spectroscopic analysis and the absolute configurations of compounds **1**–**4** were established by NOESY experiments and by chiral HPLC analyses of their acid hydrolysates. The absolute configuration of C-8 (a quaternary carbon substituted with a hydroxyl group) in **5** of preechinulin class was firstly determined by electronic circular dichroism (ECD) calculations. All these compounds were evaluatedfor brine shrimp (*Artemia salina*) lethality and nematicidal activity as well as antioxidativeand antimicrobial potency.

## 1. Introduction

Marine algicolous fungi, an ecologically defined group of marine-derived microorganisms, have recently attracted much attention for natural product researchers due to their potential to produce structurally unique and biologically active metabolites [1]. Most of the algicolous fungi distributed in the genera of *Aspergillus* and *Penicillium*, which belong to the facultative marine fungi that were frequently discovered from different groups of algae [1,2]. However, four strains of the fungal genus *Eurotium* including *E. cristatum* [3], *E. rubrum* [4], *Eurotium* sp. [5], and *E. herbariorum* [6], have been characterized from the brown alga of *Sargassum thunbergii* [3], red alga of *Asparagopsis taxiformis* [4] and *Bostrychia tenella* [5], and green alga of *Epiactis prolifera* [6]. Compared to its unknown ecological role, the fungal genus *Eurotium* has attracted more interests on its profilic source of indolediketopiperazine derivatives containing *L*-tryptophan and *L*-alanine units, generally characterized by a reverse isoprene chain at C-2 of the indole nucleus [3,7,8,9,10]. These compounds were reported to possess antibacterial [3,9], nematicidal [11], antioxidative (DPPH assay) [12,13], moderate immunosuppressive [14], and anti HIV-1 replication activities [15], as well as cytotoxic effects against P388 [12] and A-549 cells [16]. During our ongoing search for bioactive metabolites from marine-derived fungi [3,7,8,9,17,18,19,20,21], the fungal species of *Eurotium cristatum*, usually inhabiting with black tea [22], was isolated from the marine brown alga *Sargassum thunbergii* for the first time and was chemically investigated in 2012 [3]. As a result, several indole alkaloids, including two new congeners having serine residue, one new dimer, and one new derivative featuring the open ring of 2,5-diketopiperazine moiety, have been characterized and these compounds were proved to possess brine shrimp lethality and antibacterial activity against *E. coli* [3].

Further work on the same fungal strain has now led to the isolation and identification of four new indolediketopiperazine derivatives (**1**–**4**) and nine known congeners (**5**–**13**). In order to search bioactive leading compounds, these metabolites were evaluated for brine shrimp (*Artemia salina*) lethality, nematicidal, antioxidative and antimicrobial activities. This paper describes the isolation, structure elucidation, and biological activity of the isolated compounds.

## 2. Results and Discussion

### 2.1. Structural Elucidation of Indolediketopiperazines

**Figure 1:**
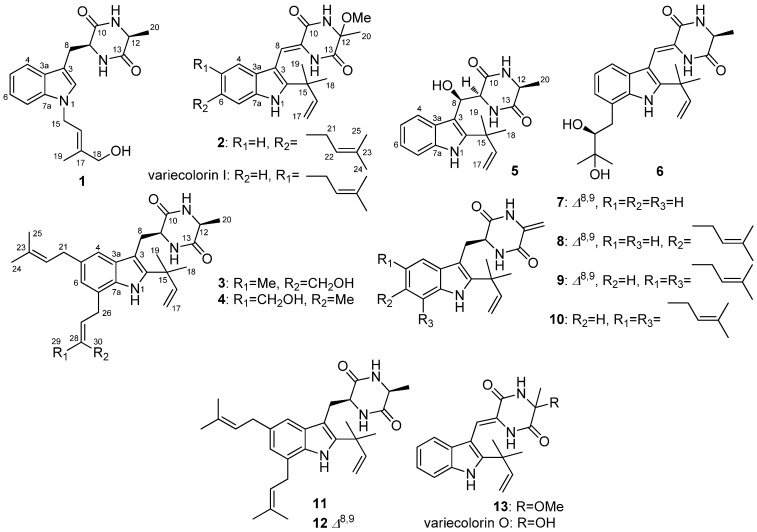
Chemical structures of compounds **1**–**13**.

The rice culture of *E. cristatum* EN-220 was exhaustively extracted with EtOAc and the extract was further then purified by a combination of column chromatography on Si gel, Sephadex LH-20, and Lobar LiChroprep RP-18 to obtain subfractions, which were further purified by semi-preparative HPLC (with Elite ODS-BP column) to yield compounds **1**–**13** (Figure 1).

**Figure 2:**
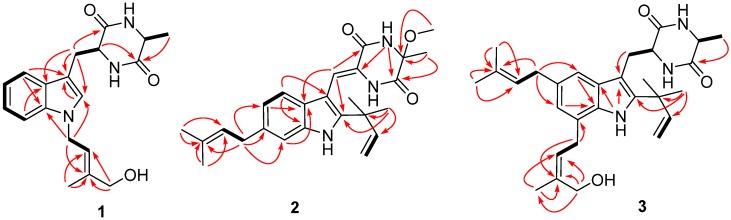
Key HMBC (arrows) and ^1^H–^1^H COSY (bold lines) correlations of compounds **1**–**3**.

**Figure 3:**
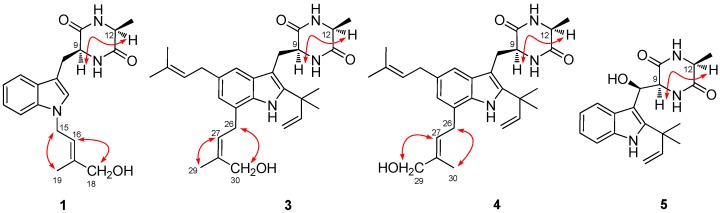
Key NOE correlations of compounds **1** and **3**–**5**.

Compound **1** was obtained as a colorless amorphous powder. The molecular formula C_19_H_23_N_3_O_3_ was assigned on the basis of HRESIMS (Figure S2 in the Electronic Supplementary Materials, ESM) and NMR data (Tables S1 and S2 in the ESM). The UV absorptions at λ_max_ 222 and 286 nm suggested the presence of an indole moiety [12]. The ^13^C NMR along with the DEPT spectroscopic data (Table S2 in the ESM) revealed the presence of 19 carbon atoms including six quaternary carbons, eight methines (with one olefinic and five aromatic), three methylenes (with one oxygenated), and two methyl groups. Detailed analysis of the NMR spectroscopic data revealed that **1** might be an indolediketopiperazine derivative similar to cyclo(alanyltryptophyl) [23]. However, additional signals atδ_H_4.80/δ_C_44.0 (CH_2_, C-15), δ_H_ 5.62/δ_C_120.2 (CH, C-16), δ_C_140.4 (C, C-17), δ_H_ 3.95/δ_C_67.5 (CH_2_, C-18), and δ_H_ 1.84/δ_C_13.9 (Me, C-19), which corresponding to an isoprenic unit with a terminal hydroxy substitution, were observed in the NMR spectra of **1** (Tables S1 and S2 in the ESM). This isoprene unit was placed on the nitrogen atom of the indole ring system as evidenced by the observed HMBC correlations from H-15 to C-2 and C-7a (Figure 2). The NOE correlations from H-16 to H-18 and from H-15 to H-19 indicated the *E*-geometry for the C-16 double bond, while the correlation from H-9 to H-12 suggested the cofacial orientation of these protons (Figure 3). Based on the above evidence, the structure of compound **1** was determined and it was named as *N*-(4′-hydroxyprenyl)-cyclo(alanyltryptophyl).

The HRESIMS data of compound **2** (Figure S8 in the ESM) demonstrated its molecular formula to be C_25_H_31_N_3_O_3_, same as that of variecolorin I (Figure 1) [12], indicating that these two compounds are isomers. Except for signals corresponding to the phenyl nucleus, the NMR data and UV absorptions of these two compounds were very similar. The aromatic proton signals at δ_H_ 7.08 (1H, d, *J* = 8.1 Hz, H-4), 6.83 (1H, d, *J* = 8.1 Hz, H-5), and 7.20 (1H, s, H-7) indicated that the isoprenic unit was connected at C-6 of the indole moiety of **2**, not at C-5 as that of variecolorin I [12]. This deduction was further supported by the HMBC correlations from H-21 to C-5, C-6, and C-7 (Figure 2). The lower-field-shifted methine proton signal for H-8 at δ_H_ 6.99 (1H, s, H-8) of **2** implied that this proton was influenced by the deshielding effect of the C=O group, which suggested the double bond at C-8 has *Z*-geometry [24]. The zero specific rotation, similar to variecolorin I [12], suggested that **2** was a racemic compound. To further confirm whether compound **2** is a natural product or an artifact due to the use of MeOH during the purification procedures, an experiment simulating the conditions that were used during chromatographic purification procedures was performed. As the diketopiperazine precursor of **2** was not available in our laboratory, the similar compound, variecolorin O (Figure 1), was used in the experiment. A mixture of variecolorin O and Si gel in the solvent CHCl_3_/MeOH (1:1, *v*/*v*) was stirred at room temperature for 72 h and the mixture was checked by HPLC every 24 h. Compound **13**, the methylated product of variecolorin O, was analyzed by HPLC at the same time. The HPLC profiles showed that variecolorin O is very stable and could not be transformed to compound **13** under the experiment conditions (Figure S26 in the ESM). This result supports that compound **2** is most likely a natural product. Based on the above evidence, the structure of **2** was determined and it was named as isovariecolorin I.

30-Hydroxyechinulin (**3**) has the molecular formula C_29_H_39_N_3_O_3_ as determined by HRESIMS (Figure S14 in the ESM). The 1D NMR data of **3** (Tables S1 and S2 in the ESM) showed marked similarities to echinulin (**11**) [23], except that one methyl group at δ_C_ 17.9 (C-30)/δ_H_ 1.80 (H-30) of echinulin disappeared in the NMR spectra of **3**. Instead, signals corresponding to an oxymethylene group at δ_C_ 60.0 (C-30)/δ_H_ 4.13 (H-30) were observed in the NMR spectra of **3**. The HMBC correlations from H-30 to C-27, C-28, and C-29 verified the structure of **3** (Figure 2). The double bond at C-27 was determined to have *Z*-geometry by the observed NOE correlations from H-27 to H-29 and from H-26 to H-30, while NOE from H-9 to H-12 indicated the *cis* relationship of this proton pair (Figure 3).

The HRESIMS of 29-hydroxyechinulin (**4**) (Figure S20 in the ESM) gave the same molecular formula C_29_H_39_N_3_O_3_ as that of **3**, and comparison the NMR data (Tables S1 and S2 in the ESM) with those of **3** revealed that compound **4** possessed the same planar structure as that of **3**. However, the *E*-geometry for the double bond at C-27, differed from that in compound **3**, was evidenced by the observed NOE correlations from H-27 to H-29 of the oxygenated CH_2_ group and from H-26 to H-30 of the methyl group (Figure 3). Same as that of **3**, H-9 and H-12 also has *cis* relationship as corroborated by the observed NOE correlation among the two protons.

**Figure 4:**
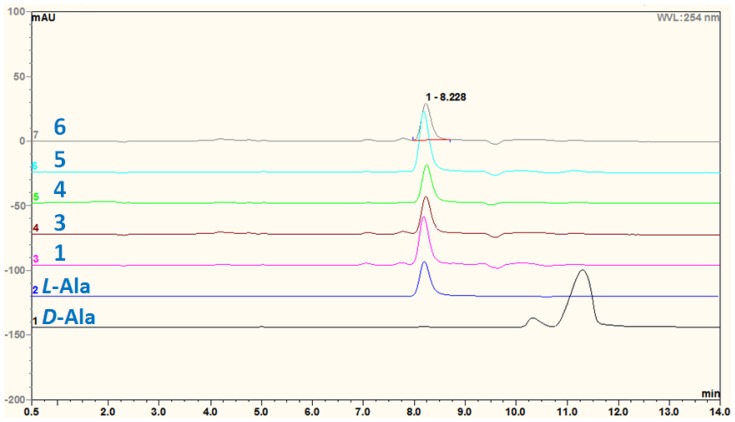
Chiral HPLC profiles of acidic hydrolysates of compounds **1** and **3**–**6** containing the alanine (Ala) residue. *Acidic hydrolysis condition*: 6 N HCl (aq.) at 110 °C for 24 h. *Chromatographic conditions*: chiral column: Phenomenex, Chirex 3126 *N*,*S*-dioctyl-(d)-penicillamine, 250 mm × 4.60 mm, 5 μm; mobile phase: 2 mM CuSO_4_; flow rate: 1 mL/min; UV detection: 254 nm; injection volume: 10 μL. Results: *l*-Ala: 8.23 min; *d*-Ala: 11.27 min.

The alanine residue in the 2,5-dikeopiperazine unit of compounds **1**, **3** and **4** was determined to have *L*-configuration by the results from the chiral HPLC analyses (Figure 4) of the acid hydrolysis products [25], as compared with that of the authentic standards, which indicated that the absolute configuration at C-12 of these compounds was *S*. The *S*-configuration at C-9 of compounds **1**, **3** and **4** was therefore deduced (Figure 3).

**Figure 5:**
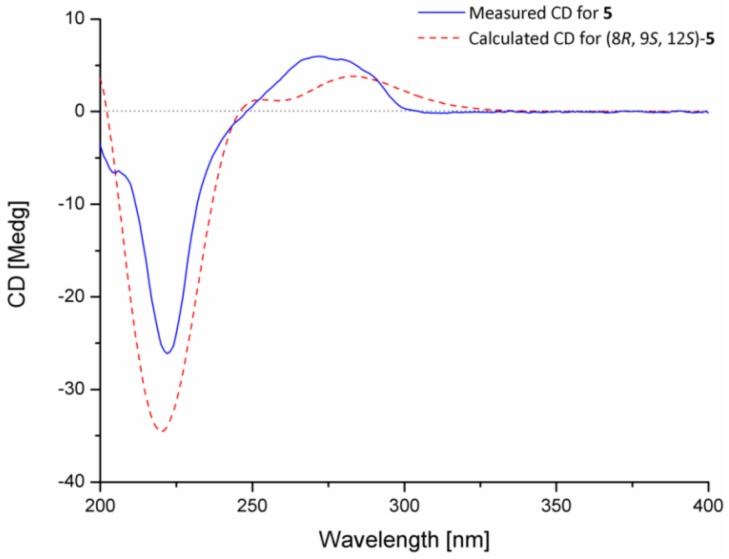
Comparison of calculated electronic circular dichroism (ECD) spectrum for (8*R*, 9*S*, 12*S*) with the experimental spectra of **5** in MeOH.

Compound **5**, an indolediketopiperazine derivative of preechinulin class, was also isolated in this study. This compound was very recently characterized from a marine-derived fungus *Eurotium rubrum* by Chen and co-workers and was named as rubrumline M (**5**) [26]. A literature searching result showed that only two indolediketopiperazine derivatives of preechinulin class, rubrumline M (**5**) and arestrictin A [27], had been isolated and characterized with a hydroxyl group substituted at C-8. However, the absolute configuration of C-8 (a quaternary carbon substituted with a hydroxyl group) in **5** as well as in arestrictin A had not been determined. To clarify the absolute configuration, the electronic circular dichroism (ECD) quantum chemical calculations in Gaussian 09 [28], was performed. To obtain minimum energy conformers, geometry optimization of each possible isomer of **5** was conducted, and the time-dependent density functional method was then used at the B3LYP/6-31G(d) level to generate calculated ECD spectra of **5**. The experimental and calculated ECD spectra for **5** showed excellent agreement for the 8*R*, 9*S*, and 12*S*-absolute configuration in **5** (Figure 5). Both the calculated and experimental data spectra showed a strong negative Cotton effect (CE) near 220 nm and positive CE around 275 nm. These close similarities enabled assignment of the absolute configuration of **5** as shown in Figure 1.

In addition to the isolation of indolediketopiperazine derivatives **1**–**5**, eight other congeners including rubrumazine B (**6**) [9], neoechinulin B (**7**) [24], neoechinulin C (**8**) [24], alkaloid E-7 (**9**) [29], didehydroechinulin (**10**) [13], echinulin (**11**) [23], dehydroechinulin (**12**) [7], and variecolorin H (**13**) [12], were also isolated and identified. The structures of these compounds were determined by detailed analysis of their spectroscopic data and by comparison with that reported in the literature.

### 2.2. Bioactivities of Indolediketopiperazines

Brine shrimp (*Artemia salina*), an aquatic species featuring with the highly sensitive to toxic and easily culturable to researchers, could be used as a model organism for a preliminary and quick screening of the insecticidal activity [30,31]. Previous research of *E. cristatum* EN-220 showed that the fermentation extract exhibited a significant lethality to brine shrimp [3]. Therefore, in order to search the insecticidal leading compounds, **1**–**13** were evaluated for the lethal activity against brine shrimp [32,33], and furthermore, nematicidal activity against *Panagrellus redivivus* [11,34] was evaluated as well (Table 1). In the brine shrimp assay, compounds **2**, **8**, **9**, and **10** showed lethal activity with LD_50_ values of 19.4, 70.1, 19.8, and 27.1 μg/mL, respectively, while compounds **3**, **4**, and **7** exhibited weaklethal activities. The nematicidal assay showed that compounds **2**, **9**, and **10** exhibited weak activity with LD_50_ values of 110.3, 106.7, and 126.4 μg/mL, respectively. Compounds **9** and **10** were notablely more active against brine shrimp and *Panagrellus redivivus* than **7** and **8**, which was probably due to the number and position of the isoprenic unit. This deduction was also proved by the structure differences between compounds **2** and **13** compared with their activities. The insecticidal activities of compounds **9** and **10** might also be related to the exocyclic double bond in the 2,5-diketopiperazine moiety compared to the structures of **11** and **12**, which did not show any activity.

Based on the reported typical bioactivity of indolediketopiperazines, the isolated compounds were further assessed for antioxidative and antimicrobial activities, with the purpose of enriching the bioactive diversity of these compounds. Compounds **2** and **6**–**13** were evaluated for antioxidative activities against DPPH and superoxide anion radicals [8,9] (Table 2). Compound **12** showed potent radical scavenging activity against DPPH with IC_50_ value of 6.4 μg/mL, which was comparable to that of the positive control ascorbic acid (IC_50_ 2.0 μg/mL). The other compounds (**2**, **6**–**12**) exhibited moderate antioxidative activities with IC_50_ values ranging from 10.1 μg/mL to 28.5 μg/mL. However, all the tested compounds did not show the superoxide anion radical scavenging activity.

Compounds **1**–**13** were also evaluated for the antimicrobial activities [3,9] against six pathogenic bacteria (*Escherichia coli*, *Staphyloccocus aureus*, *Bacillus subtilis*, *Micrococcus luteus*, *Salmonella enteric*, and *Bacillus pumilus*) and nine plant-pathogenic fungi (*Alternaria brassicae*, *Valsa mali*, *Physalospora obtuse*, *Alternaria solania*, *Sclerotinia miyabeana*, *Magnaporthe grisea*, *Fusarium oxysporium*, *Botryosphaeria dothidea*, and *Colletotrichum gloeosporioides*). Compounds **11**, **12**, and **13** showed weak activity against *S. aureus* with the same MIC value of 256 μg/mL, while compound **6** exhibited moderate activity against *Magnaporthe grisea* with the MIC value of 64 μg/mL.

Indole diketopiperazine alkaloids are characterized by condensation of the tryptophan with a second amino acid such as *L*-alanine, *L*-proline, or *L*-tryptophan, forming the ring of diketopiperazine unit [10]. The isolated compounds of *E. cristatum* EN-220, featuring with the second amino acid of *L*-alanine, showed potent brine shrimp lethality and antioxidative activity against DPPH. However, the isolated compounds did not exhibit any antimicrobial activities, which indicated that indolediketopiperazines having alanine residue might have the low relevance to the antimicrobial activity. Bioactive compounds with potent brine shrimp lethality also showed weak nematicidal activity, further proving the application of brine shrimp as a preliminary screening of insecticidal activity.

## 3. Experimental Section

### 3.1. General Procedures

Optical rotations were obtained on a Jasco P-1020 digital polarimeter (Jasco Corporation, hachioji-shi, Tokyo, Japan). UV spectra were measured on a PuXi TU-1810 UV-visible spectrophotometer (Beijing Puxi General Instrument Corporation, Pinggu, Beijing, China). ECD spectra were acquired on a Chirascan spectropolarimeter (Applied Photophysics Ltd., Surrey, UK). 1D and 2D NMR spectra were recorded at 500 MHz and 125 MHz for ^1^H and ^13^C, respectively, on a Bruker Avance 500 spectrometer (Bruker Biospin Group, Karlsruhe, Germany) with TMS as internal standard. Mass spectra were determined on a VG Autospec 3000 (VG Instruments, London, UK) or an API QSTAR Pulsar 1 mass spectrometer (Applied Biosystems, Foster, Waltham, MA, USA). Column chromatography (CC) was performed with Si gel (200–300 mesh, Qingdao Haiyang Chemical Co., Qingdao, Shandong, China), Lobar LiChroprep RP-18 (40–63 μm; Merck, Kenilworth, NJ, USA), and Sephadex LH–20 (18–110 μm, Merck). Semi-preparative HPLC was performed using a Dionex HPLC system equipped with a P680 pump, an ASI-100 automated sample injector, and a UVD340U multiple wavelength detector controlled using Chromeleon software, version 6.80 (Dionex Corporation, Sunnyvale, CA, USA).

### 3.2. Fungal Material

The endophytic fungus *Eurotium cristatum* EN-220 was isolated from the marine alga *Sargassum thunbergii* collected from the coast of Qingdao, China, in November 2009. The fungus was identified by analysis of the ITS region of the rDNA, as described in our previous report [3]. The sequence data derived from the fungal strain was deposited at GenBank, with accession No. JQ743649. The strain is preserved at the Institute of Oceanology, Chinese Academy of Sciences.

### 3.3. Fermentation, Extraction, and Isolation

For chemical investigations, the fungal strain was statically fermented at r.t. for 30 days on sterilized solid medium containing rice (100 g/flask), peptone (0.6 g/flask), and sea water (100 mL/flask) in 1-L Fernbach flasks (×100). The rice culture of the fungal strain was exhaustively extracted with EtOAc to give a crude extract, which was dried and fractionated by Si gel vacuum liquid chromatography (VLC) using different solvents of increasing polarity from petroleum ether (PE) to MeOH to yield 12 fractions (Fractions (Frs.) 1–12) based on TLC analysis.

Fr. 6 was purified by column chromatography (CC) over Si gel eluting with a PE–acetone gradient (from 30:1 to 5:1) and by semi-preparative HPLC (Elite ODS-BP column, 10 μm; 10.0 × 300 mm; 70% MeOH/H_2_O, 3 mL/min) to afford **9** (6.2 mg, *t_R_* 17.1 min) and **10** (8.8 mg, t*_R_* 15.7 min). Fr. 7 was purified by CC over Si gel eluting with a CHCl_3_–MeOH gradient (from 100:1 to 20:1) to afford two subfrations (Fr. 7-1 and Fr. 7-2). Fr. 7-1 was further purified by CC over RP-18 eluting with a MeOH–H_2_O gradient (from 1:9 to 1:0) and by semi-preparative HPLC (from 65% to 85% MeOH/H_2_O, 3 mL/min) to afford **2** (11.5 mg, *t_R_* 17.8 min), **7** (14.8 mg, *t_R_* 13.2 min), **8** (19.2 mg, *t_R_* 19.2 min), **11** (40.2 mg, *t_R_* 24.5 min), **12** (18.8 mg, *t_R_* 26.8 min), and **13** (6.8 mg, *t_R_* 12.1 min). Fr. 7-2 was further purified by Sephadex LH-20 (MeOH) and by semi-preparative HPLC (65% MeOH/H_2_O, 3 mL/min) to afford **6** (3.1 mg, *t_R_* 15.3 min). Fr. 9 was purified by CC over RP-18 eluting with a MeOH–H_2_O gradient (from 1:9 to 1:0) to afford two subfractions (Fr. 9-1 and Fr. 9-2). Fr. 9-1 was further purified by semi-preparative HPLC (45% MeOH/H_2_O, 3 mL/min) to afford **1** (5.9 mg, *t_R_* 16.8 min) and **5** (3.9 mg, *t_R_* 11.1 min). Fr. 9-2 was further purified by semi-preparative HPLC (65% MeOH/H_2_O, 3 mL/min) to afford **3** (5.8 mg, *t_R_* 17.4 min) and **4** (3.1 mg, *t_R_* 18.9 min).

Detailed ^1^H and ^13^C NMR data of compounds **1**–**4** were shown in Tables S1 and S2 of the ESM, and the main physical and chemical properties could also be viewed in Table S3 of the ESM.

### 3.4. ECD Calculation of Compound ***5***

Conformational searches for **5** were performed via the molecular mechanics using MM + method in HyperChem 8.0 software (Beijing HuanZhongRuiChi Technology Co., Ltd., Daxing, Beijing, China), and the geometries were further optimized at B3LYP/6-31G(d) level via Gaussian 09 software (Gaussian Inc., Wallingford, CT, USA) to give the energy-minimized conformers. Then, the optimized conformers were subjected to the calculations of ECD spectra using TD-DFT at B3LYP/6-31G(d) level; solvent effects of the MeOH solution were evaluated at the same DFT level using the SCRF/PCM method [28].

### 3.5. Acidic Hydrolysis of Compounds ***1*** and ***3***–***6***

Compound **1** (1 mg) was dissolved in 3 mL of 6 N HCl and heated in a sealed tube at 110 °C for 24 h. The hydrolysate was dried, reconstituted in H_2_O (1 mL), and then subjected to chiral HPLC analysis. The retention times of the authentic amino acids were as follows: *d*/*l*-Ala, t*_R_* 11.27/8.23 min. The retention time of amino acid in hydrolysate of **1** was 8.23 min, indicating that the amino acid in **1** was *l*-Ala. By the same procedure, compounds **3**–**6** gave the same results that the amino acids in these compounds were all *l*-Ala.

### 3.6. Verification of Compound ***2*** Being Not an Artifact

As the probable oxidized diketopiperazine precursor of **2** was not isolated in our experiment, the similar compound, variecolorin H (**13**), as well as its oxidized precursor, variecolorin O [12], were used in the experiment. A sample of variecolorin O (2 mg) was mixed with Si gel (1 g) in 5 mL solvent (CHCl_3_:MeOH = 1:1, *v*/*v*) and the mixture was stirred at r.t. for 72 h. The mixture was analyzed using HPLC every 24 h and the retention time was consistent to variecolorin O with 28.5 min, while variecolorin H with 30.9 min.

### 3.7. Bioassay

Evaluation of brine shrimp (*Artemia salina*) toxicity [32,33], nematicidal [11,34], and antioxidative activities [12,13] were performed as previously reported. Antimicrobial assays against six bacteria (*S. aureus*, *E. coli*, *B. subtilis*, *M. luteus*, *S. enteric*, and *B. pumilus*) and nine plant-pathogenic fungi (*A. brassicae*, *V. mali*, *P. obtuse*, *A. solania*, *S. miyabeana*, *M. grisea*, *F. oxysporium*, *B. dothidea*, and *C. gloeosporioides*) were carried out using the broth microdilution method [3,9]. Chloramphenicol and amphotericin B were used as positive controls against bacteria and fungi, respectively.

## 4. Conclusions

Thirteen indolediketopiperazine derivatives (**1**–**13**), including four new compounds (**1**–**4**), were isolated and identified from the culture extract of the marine algal-derived endophytic fungus *Eurotium cristatum* EN-220. The relative and absolute configurations of compounds **1**–**4** were established by NOESY experiments and chiral HPLC analyses of their acid hydrolysates. The absolute configuration of 8-quarternary carbon substituted with a hydroxyl group in **5** of preechinulin class was firstly determined by ECD calculations. Compounds **2**, **9**, and **10** exhibited potent lethal activity against brine shrimp and weak nematicidal effect against *Panagrellus redivivus*. This is the first report for the brine shrimp inhibition activity of compounds **2**, **9**, and **10**. In addition, compound **12** showed potent radical scavenging activity against DPPH radicals.

## Figures and Tables

**Table 1:** Brine shrimp lethality and nematicidal activity of compounds **1**–**13** (LD_50_, μg/mL).

Compd.	1	2	3	4	5	6	7	8	9	10	11	12	13
brine shrimp lethality	n.a.	19.4	138.1	140.6	n.a.	n.a.	105.2	70.1	19.8	27.1	n.a.	n.a.	n.a.
nematicidal activity	n.a.	110.3	n.a.	n.a.	n.a.	n.a.	>200	>200	106.7	126.4	n.a.	n.a.	n.a.

n.a.: no activity.

**Table 2:** DPPH radical scavenging activity of compounds **2** and **6**–**13** (IC_50_, μg/mL).

Compd.	2	6	7	8	9	10	11	12	13	Ascorbic Acid
IC_50_	20.6	28.5	10.9	12.1	10.1	13.3	13.8	6.4	18.7	2.0

## Supplementary Materials

The following are available online at www.mdpi.com/1660-3397/15/2/24/s1, 1D and 2D NMR spectra and synthetic tables of the NMR data of compounds **l**–**4** including their physical properties and HPLC profiles for checking the artificial nature of variecolorin O.
