# Supplementary material for: Indolediketopiperazine Alkaloids from Eurotium cristatum EN-220, an Endophytic Fungus Isolated from the Marine Alga Sargassum thunbergii
Source: Mar Drugs. 2017 Jan 25;15(2):24. doi: 10.3390/md15020024 (PMC5334605; doi:10.3390/md15020024)
Supplement: Supplementary file 1 [file marinedrugs-15-00024-s001.docx]

Supplementary Materials: Indolediketopiperazine Alkaloids from *Eurotium cristatum* EN-220,
an Endophytic Fungus Isolated from the
Marine Alga *Sargassum thunbergii*

Feng-Yu Du, Xin Li, Xiao-Ming Li, Li-Wei Zhu and Bin-Gui Wang

Contents

| Figure S1. Chemical structures of compounds **1**–**13** and variecolorin O; | S2 |
| --- | --- |
| Table S1. ^1^H NMR data for compounds **1**–**4** (500 MHz, *J* in Hz); | S2 |
| Table S2. ^13^C NMR data for compounds **1**–**4** (125 MHz); | S3 |
| Table S3. Main physical and chemical properties parameters of compounds **1**–**4** | S3 |
| Figure S2. HR-ESI-MS spectrum of compound (**1**); | S4 |
| Figure S3. ^1^H-NMR (500 MHz, acetone-*d*_6_) spectrum of compound (**1**); | S5 |
| Figure S4. DEPT spectra of compound (**1**); | S6 |
| Figure S5. ^1^H–^1^H COSY spectrum of compound (**1**); | S7 |
| Figure S6. HSQC spectrum of compound (**1**); | S8 |
| Figure S7. HMBC spectrum of compound (**1**); | S9 |
| Figure S8. HR-ESI-MS spectrum of compound (**2**); | S10 |
| Figure S9. ^1^H-NMR (500 MHz, DMSO-*d*_6_) spectrum of compound (**2**); | S11 |
| Figure S10. DEPT spectra of compound (**2**); | S12 |
| Figure S11. ^1^H–^1^H COSY spectrum of compound (**2**); | S13 |
| Figure S12. HSQC spectrum of compound (**2**); | S14 |
| Figure S13. HMBC spectrum of compound (**2**); | S15 |
| Figure S14. HR-ESI-MS spectrum of compound (**3**); | S17 |
| Figure S15. ^1^H-NMR (500 MHz, DMSO-*d*_6_) spectrum of compound (**3**); | S18 |
| Figure S16. DEPT spectra of compound (**3**); | S19 |
| Figure S17. ^1^H–^1^H COSY spectrum of compound (**3**); | S20 |
| Figure S18. HSQC spectrum of compound (**3**); | S21 |
| Figure S19. HMBC spectrum of compound (**3**); | S22 |
| Figure S20. ESI-MS spectrum and HR-ESI-MS data of compound (**4**); | S24 |
| Figure S21. ^1^H-NMR (500 MHz, CDCl_3_) spectrum of compound (**4**); | S25 |
| Figure S22. DEPT spectra of compound (**4**); | S26 |
| Figure S23. ^1^H–^1^H COSY spectrum of compound (**4**); | S27 |
| Figure S24. HSQC spectrum of compound (**4**); | S28 |
| Figure S25. HMBC spectrum of compound (**4**); | S29 |
| Figure S26. HPLC profiles of the mixture of variecolorin O and Si gel in solvent (CHCl_3_:MeOH = 1:1). | S30 |

**Figure S1.** Chemical structures of compounds **1**–**13** and variecolorin O.

**Table S1.** ^1^H NMR data for compounds **1**–**4** (500 MHz, *J* in Hz).

| **Position** | **1 ^[a]^** | **2 ^[b]^** | **3 ^[b]^** | **4 ^[c]^** |
| --- | --- | --- | --- | --- |
| 1-NH |  | 11.01, br s | 9.90, br s | 7.92, br s |
| 2 | 7.13, s |  |  |  |
| 4 | 7.62, d (8.0) | 7.08, d (8.1) | 7.42, s | 7.15, s |
| 5 | 7.00, t (7.8) | 6.83, d (8.1) |  |  |
| 6 | 7.12, t (7.8) |  | 7.06, s | 6.82, s |
| 7 | 7.36, d (8.2) | 7.20, s |  |  |
| 8 | 3.32, dd (14.6, 8.5)  3.24, dd (14.6, 4.1) | 6.99, s | 3.32, dd (14.4, 4.8)  3.01, dd (14.4, 9.5) | 3.66, m  3.19, m |
| 9 | 4.25, br s |  | 3.92, m | 4.40, m |
| 11-NH |  | 9.06, br s | 8.16, br s | 6.09, br s |
| 12 | 3.81, m |  | 3.81, m | 4.10, m |
| 12-OMe |  | 3.22, s |  |  |
| 14-NH |  | 9.10, br s | 7.42, br s | 5.66, br s |
| 15 | 4.80, d (6.9) |  |  |  |
| 16 | 5.62, t (6.9) | 6.06, dd (17.3, 10.6) | 6.19, dd (17.5, 10.6) | 6.11, dd (17.5, 10.6) |
| 17 |  | 5.04, d (10.6)  5.01, d (17.3) | 5.06, d (17.5)  5.02, d (10.6) | 5.16, d (17.5)  5.16, d (10.6) |
| 18 | 3.95, s | 1.44, s | 1.49, s | 1.52, s |
| 19 | 1.84, s | 1.47, s | 1.47, s | 1.52, s |
| 20 | 0.71, d (6.9) | 1.48, s | 1.33, d (7.1) | 1.53, d (7.1) |
| 21 |  | 3.37, d (7.3) | 3.29, d (7.2) | 3.39, d (7.1) |
| 22 |  | 5.33, t (7.3) | 5.30, t (7.2) | 5.35, t (7.1) |
| 24 |  | 1.71, s | 1.69, s | 1.74, s |
| 25 |  | 1.71, s | 1.69, s | 1.74, s |
| 26 |  |  | 3.61, d (7.3) | 3.58, d (7.3) |
| 27 |  |  | 5.35, t (7.3) | 5.70, t (7.3) |
| 29 |  |  | 1.74, s | 4.09, s |
| 30 |  |  | 4.13, s | 1.92, s |
| 30-OH |  |  | 5.20, br s |  |

^[a]^ Recorded in acetone-*d*_6_; ^[b]^ Recorded in DMSO-*d*_6_; ^[c]^ Recorded in CDCl_3_.

**Table S2.** ^13^C NMR data for compounds **1**–**4** (125 MHz).

| **Position** | **1 ^[a]^** | **2 ^[b]^** | **3 ^[b]^** | **4 ^[c]^** |
| --- | --- | --- | --- | --- |
| 2 | 128.6, CH | 143.9, C | 141.3, C | 141.6, C |
| 3 | 109.6, C | 103.2, C | 104.9, C | 104.4, C |
| 3a | 129.6, C | 124.1, C | 129.2, C | 129.2, C |
| 4 | 120.2, CH | 118.7, CH | 114.8, CH | 115.3, CH |
| 5 | 119.7, CH | 120.3, CH | 131.5, C | 134.1, C |
| 6 | 122.2, CH | 134.0, C | 121.4, CH | 123.0, CH |
| 7 | 110.4, CH | 110.6, CH | 122.9, C | 122.7, C |
| 7a | 137.3, C | 135.5, C | 132.1, C | 132.1, C |
| 8 | 30.2, CH_2_ | 112.2, CH | 31.1, CH_2_ | 29.4, CH_2_ |
| 9 | 56.8, CH | 124.2, C | 55.5, CH | 54.7, CH |
| 10 | 167.9, C | 161.1, C | 167.3, C | 167.8, C |
| 12 | 51.5, CH | 83.9, C | 50.2, CH | 50.9, CH |
| 12-OMe |  | 50.0, CH_3_ |  |  |
| 13 | 169.0, C | 163.2, C | 167.8, C | 168.3, C |
| 15 | 44.0, CH_2_ | 38.9, C | 38.8, C | 39.1, C |
| 16 | 120.2, CH | 145.2, CH | 146.6, CH | 145.9, CH |
| 17 | 140.4, C | 111.5, CH_2_ | 110.8, CH_2_ | 112.4, CH_2_ |
| 18 | 67.5, CH_2_ | 27.4, CH_3_ | 27.9, CH_3_ | 28.0, CH_3_ |
| 19 | 13.9, CH_3_ | 27.7, CH_3_ | 27.9, CH_3_ | 28.0, CH_3_ |
| 20 | 20.3, CH_3_ | 22.1, CH_3_ | 20.6, CH_3_ | 19.9, CH_3_ |
| 21 |  | 33.8, CH_2_ | 34.0, CH_2_ | 34.6, CH_2_ |
| 22 |  | 124.1, CH | 124.7, CH | 124.4, CH |
| 23 |  | 130.9, C | 130.2, C | 131.7, C |
| 24 |  | 25.4, CH_3_ | 25.4, CH_3_ | 25.7, CH_3_ |
| 25 |  | 17.5, CH_3_ | 17.6, CH_3_ | 17.9, CH_3_ |
| 26 |  |  | 29.5, CH_2_ | 30.6, CH_2_ |
| 27 |  |  | 125.0, CH | 123.9, CH |
| 28 |  |  | 135.0, C | 136.2, C |
| 29 |  |  | 21.7, CH_3_ | 68.6, CH_2_ |
| 30 |  |  | 60.0, CH_2_ | 13.9, CH_3_ |

^[a]^ Recorded in acetone-*d*_6_; ^[b]^ Recorded in DMSO-*d*_6_; ^[c]^ Recorded in CDCl_3_.

**Table S3.** Main physical and chemical properties of compounds **1**–**4**.

|  | **1** | **2** | **3** | **4** |
| --- | --- | --- | --- | --- |
| **Color and State** | **Colorless Amorphous Powder** | **Yellow Amorphous Powder** | **Colorless Amorphous Powder** | **Colorless Amorphous Powder** |
| ${[\alpha]}_{D}^{20}$ | +6.0  (*c* 0.50, MeOH) | 0  (*c* 0.55, MeOH) | −5.9  (*c* 0.17, CHCl_3_) | −25.7  (*c* 0.35, CHCl_3_) |
| UV (MeOH) λmax (log ε) | 222 (4.38),  286 (3.54) nm | 228 (4.51), 262 (4.02),  288 (3.95), 355 (3.98) nm | 229 (4.25),  280 (3.59) nm | 229 (4.51),  278 (3.86) nm |
| ESIMS *m*/*z*  [M + Na]^+^ | 364 | 444 | 500 | 500 |
| HRESIMS *m*/*z* [M + Na]^+^ | 364.1633  (calcd for C_19_H_23_N_3_O_3_Na^+^, 364.1637) | 444.2250  (calcd for  C_25_H_31_N_3_O_3_Na^+^, 444.2258) | 500.2885  (calcd for C_29_H_39_N_3_O_3_Na^+^, 500.2889) | 500.2887  (calcd for C_29_H_39_N_3_O_3_Na^+^, 500.2889) |
| molecular formula | C_19_H_23_N_3_O_3_ | C_25_H_31_N_3_O_3_ | C_29_H_39_N_3_O_3_ | C_29_H_39_N_3_O_3_ |


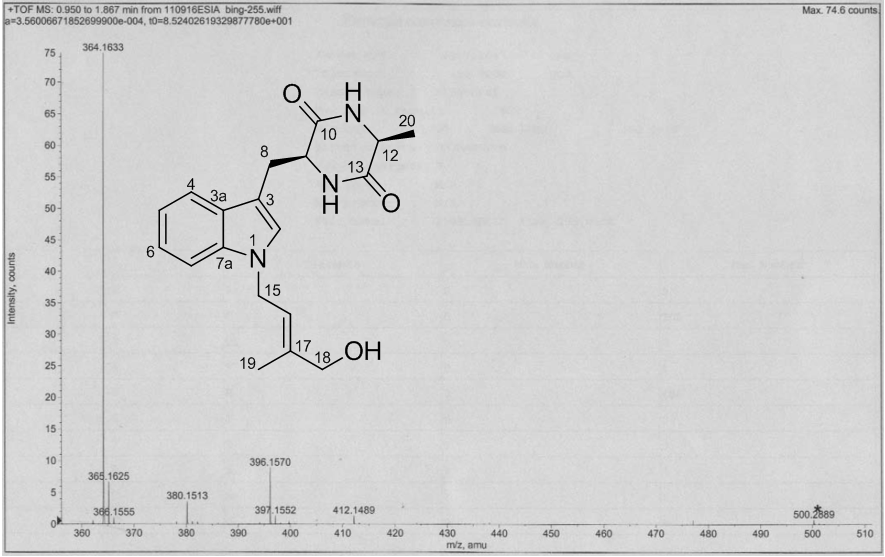


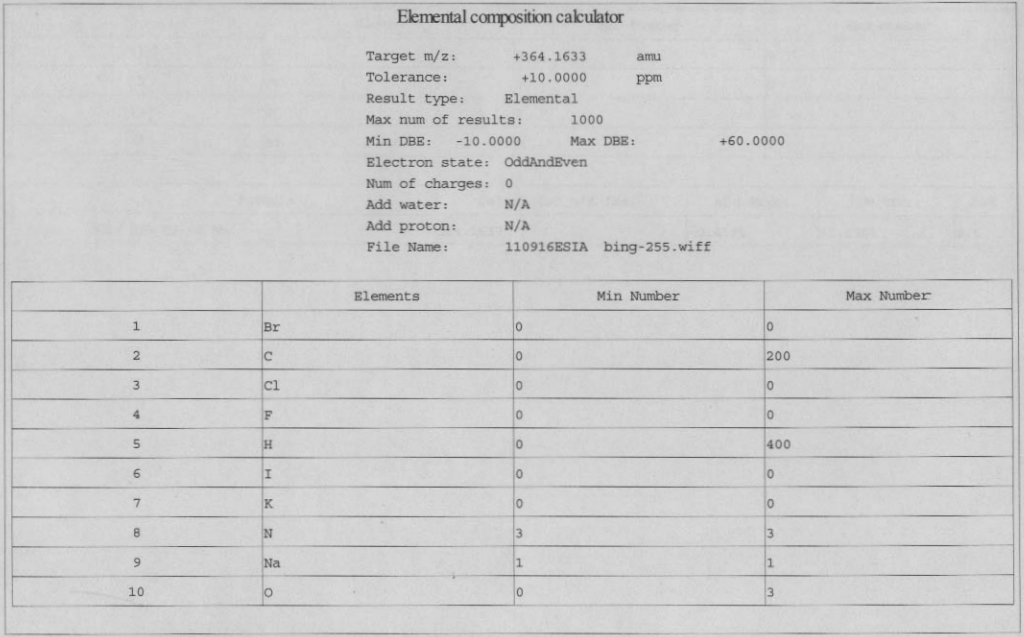


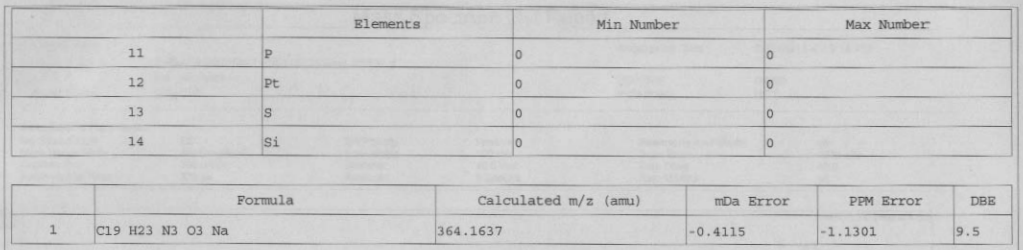


**Figure S2.** HR-ESI-MS spectrum of compound (**1**).


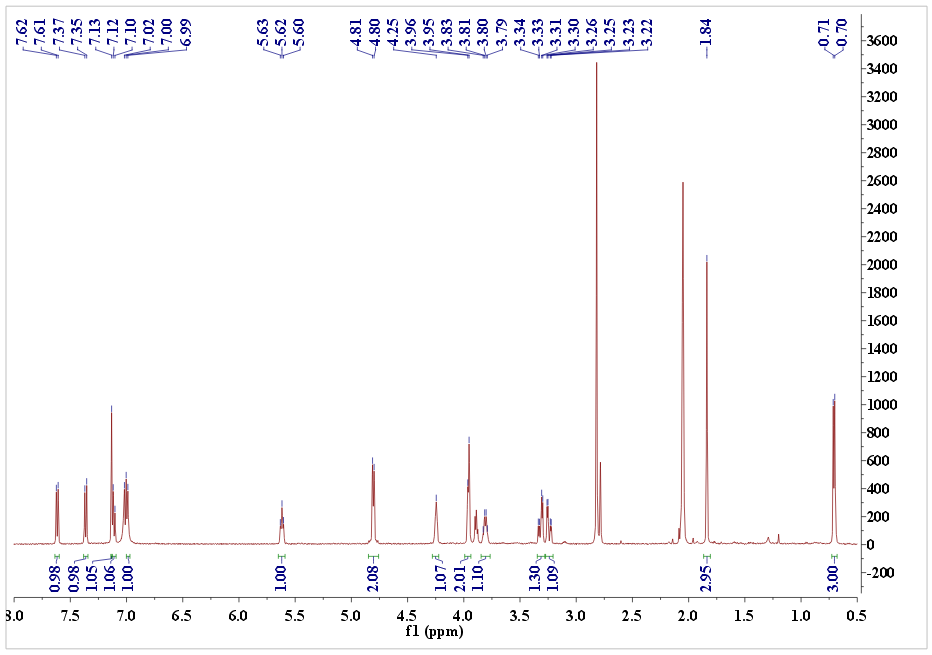

**Figure S3.** ^1^H-NMR (500 MHz, acetone-*d*_6_) spectrum of compound (**1**).


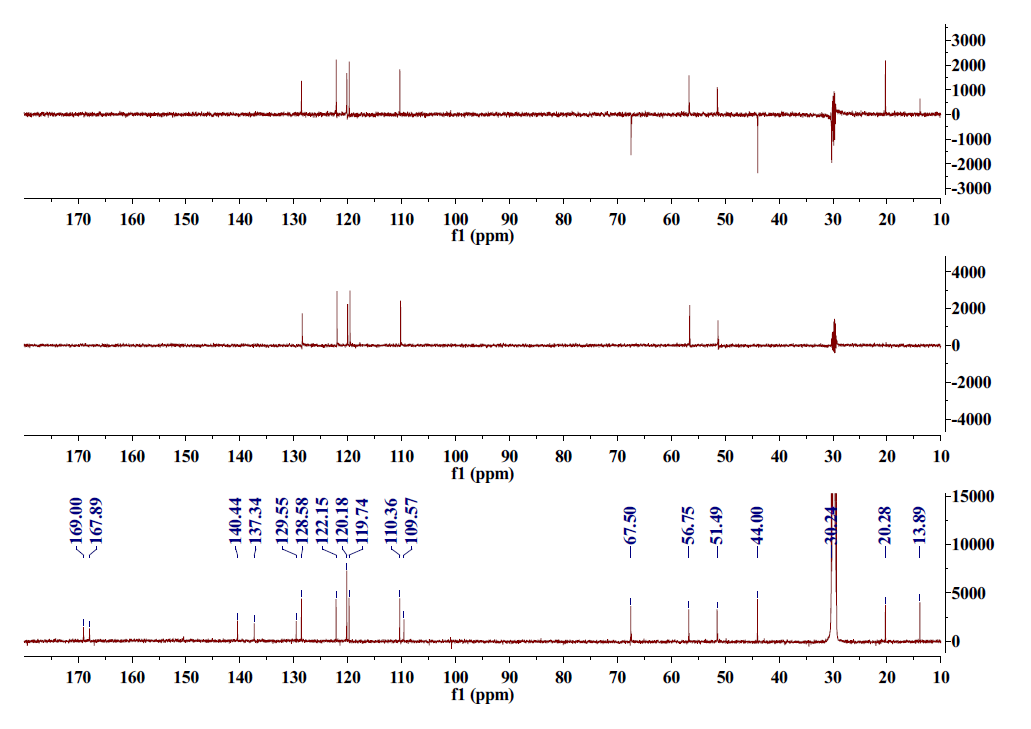


**Figure S4.** DEPT spectra of compound (**1**).


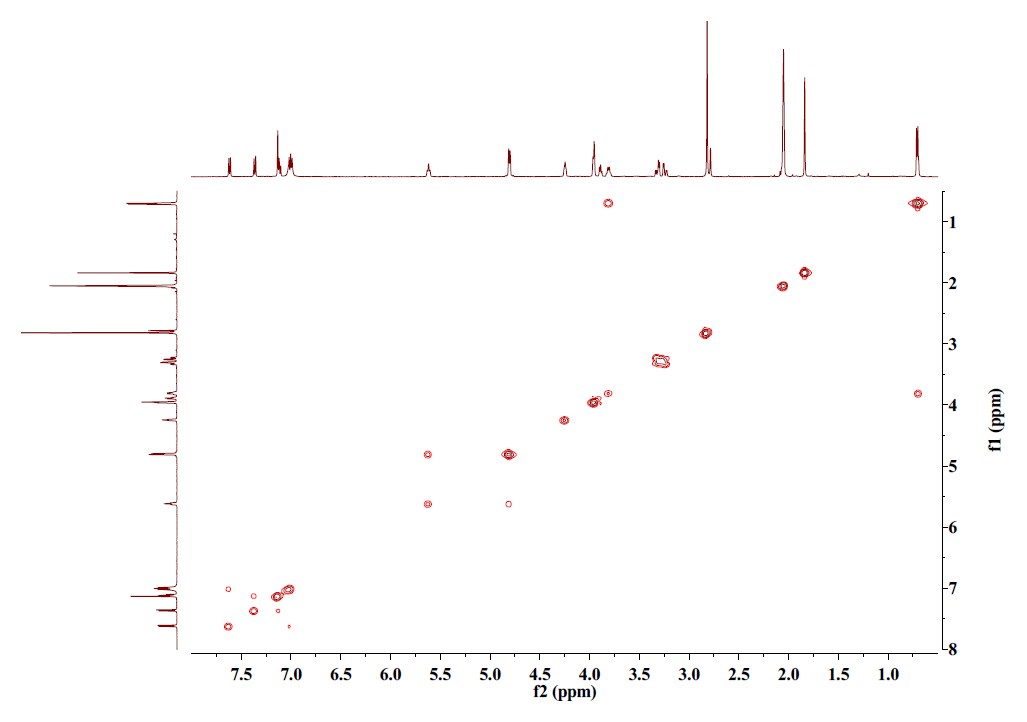


**Figure S5.** ^1^H–^1^H COSY spectrum of compound (**1**).


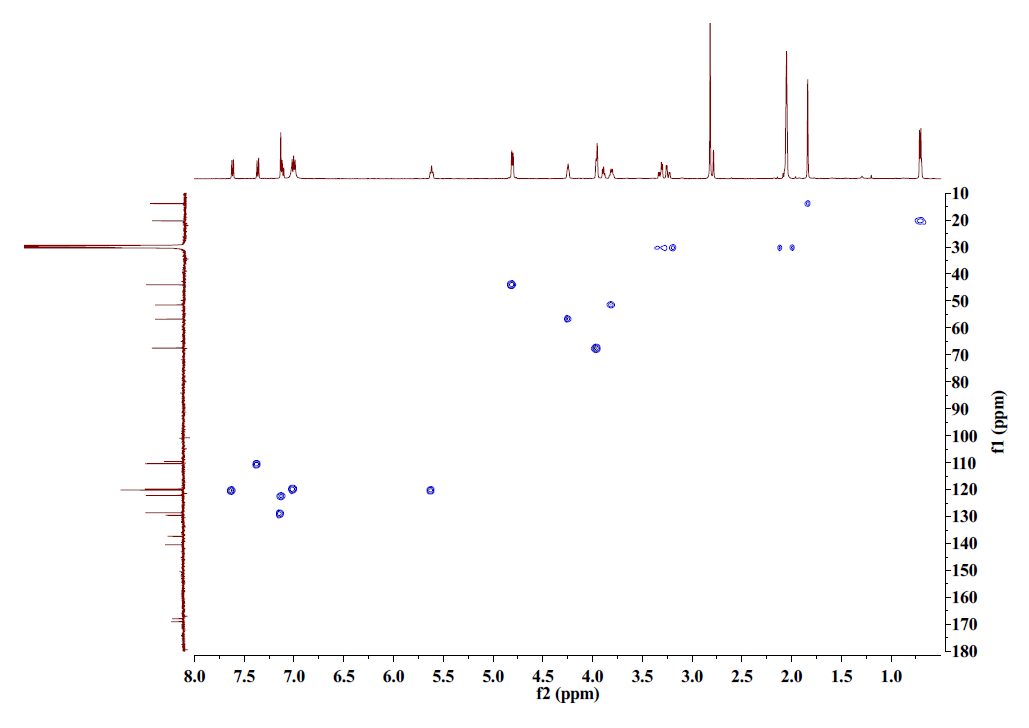


**Figure S6.** HSQC spectrum of compound (**1**).


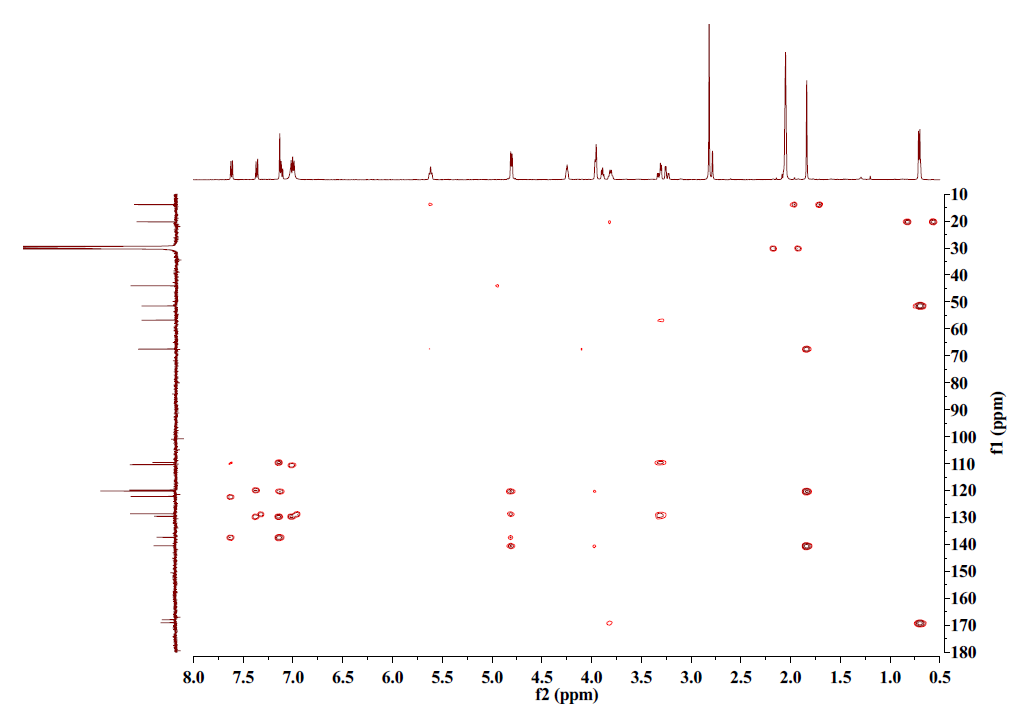


**Figure S7.** HMBC spectrum of compound (**1**).


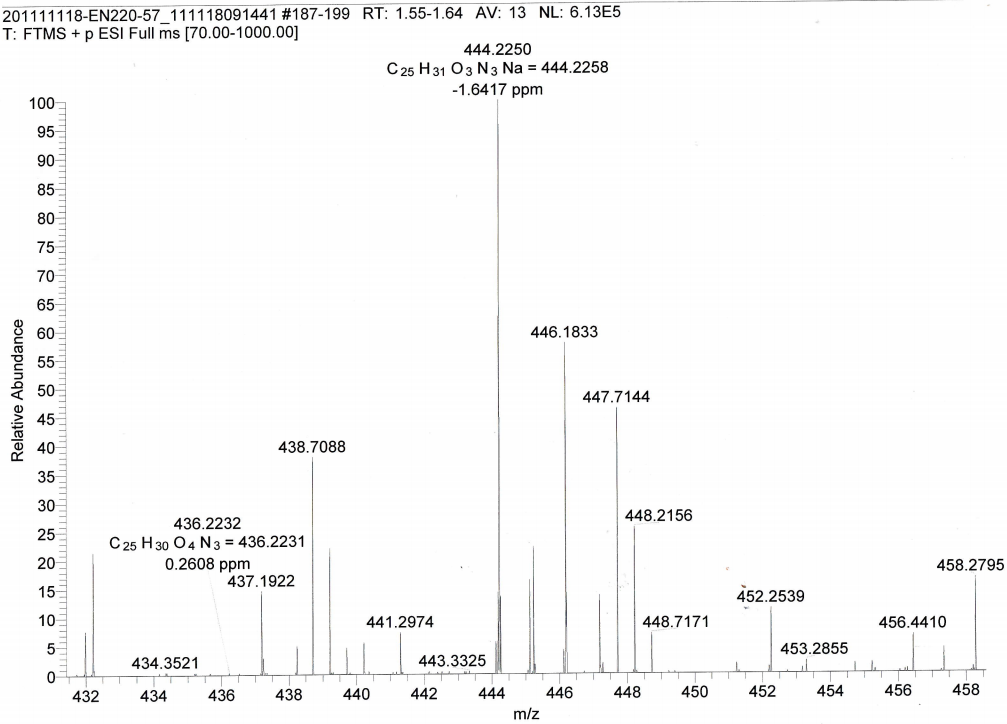


**Figure S8.** HR-ESI-MS spectrum of compound (**2**).


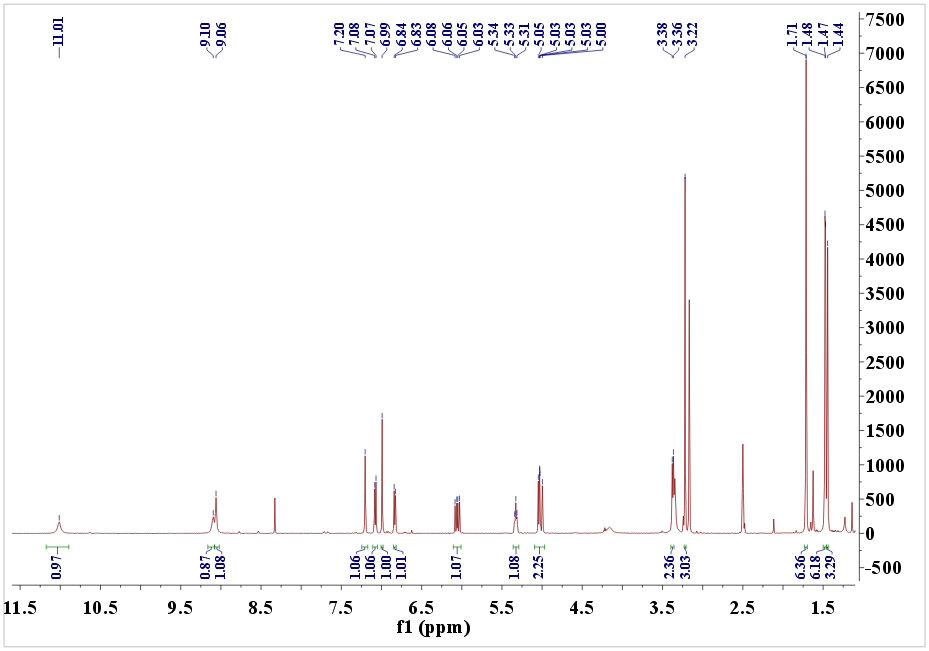

**Figure S9.** ^1^H-NMR (500 MHz, DMSO-*d_6_*) spectrum of compound (**2**).


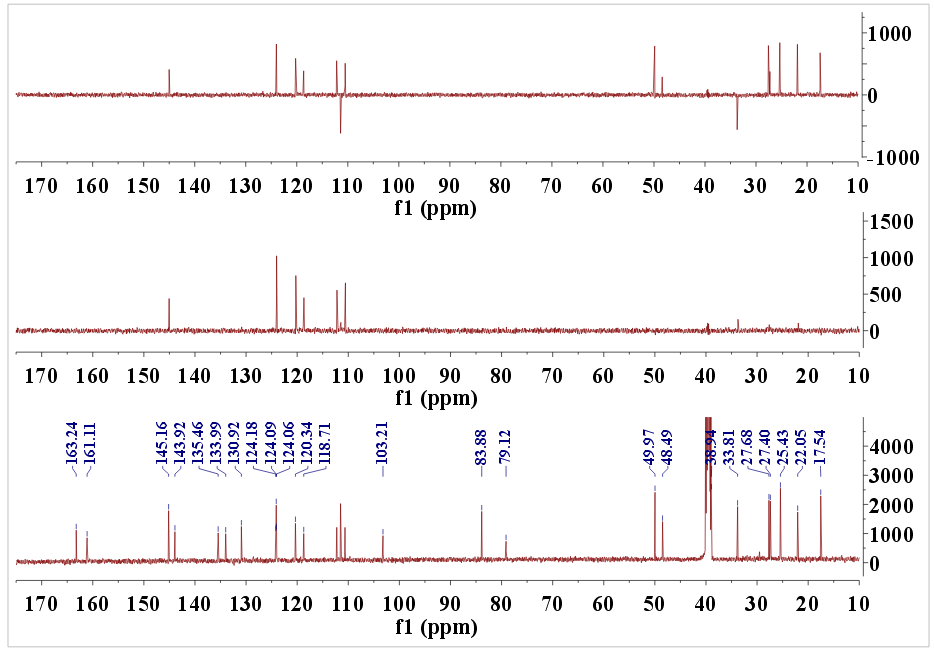


**Figure S10.** DEPT spectra of compound (**2**)


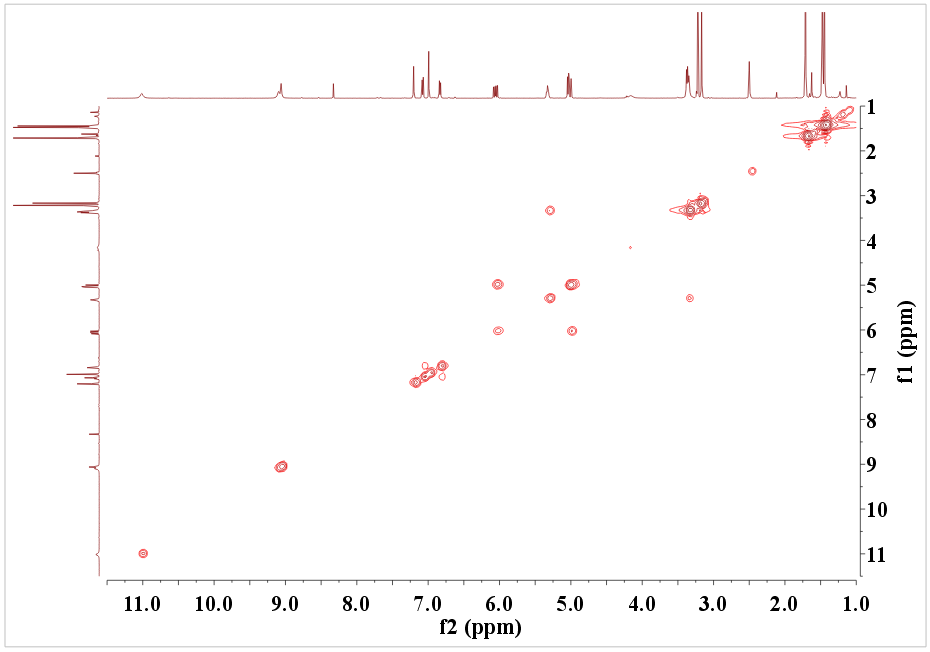


**Figure S11.** ^1^H–^1^H COSY spectrum of compound (**2**).


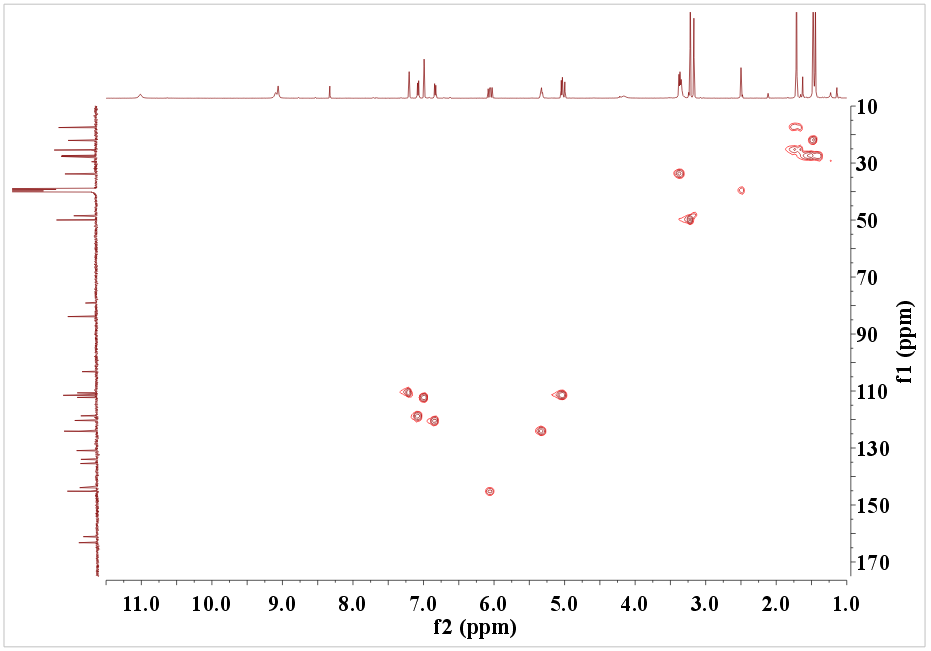


**Figure S12.** HSQC spectrum of compound (**2**).


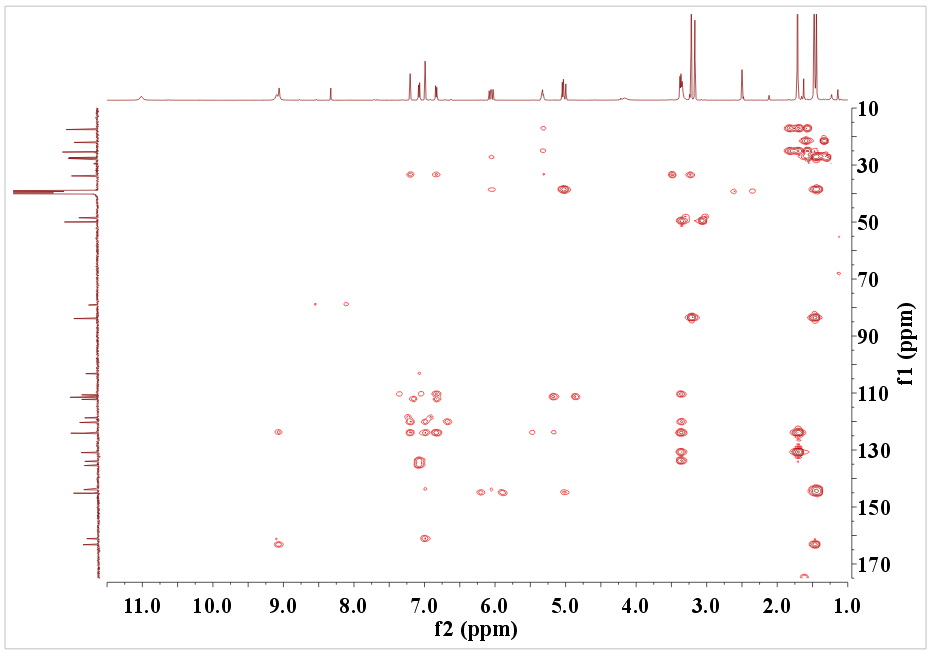


**Figure S13.** HMBC spectrum of compound (**2**).


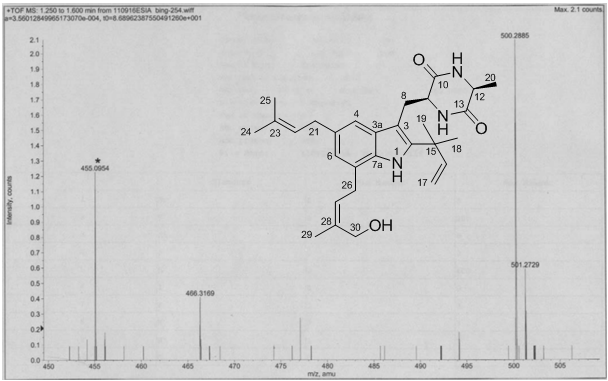


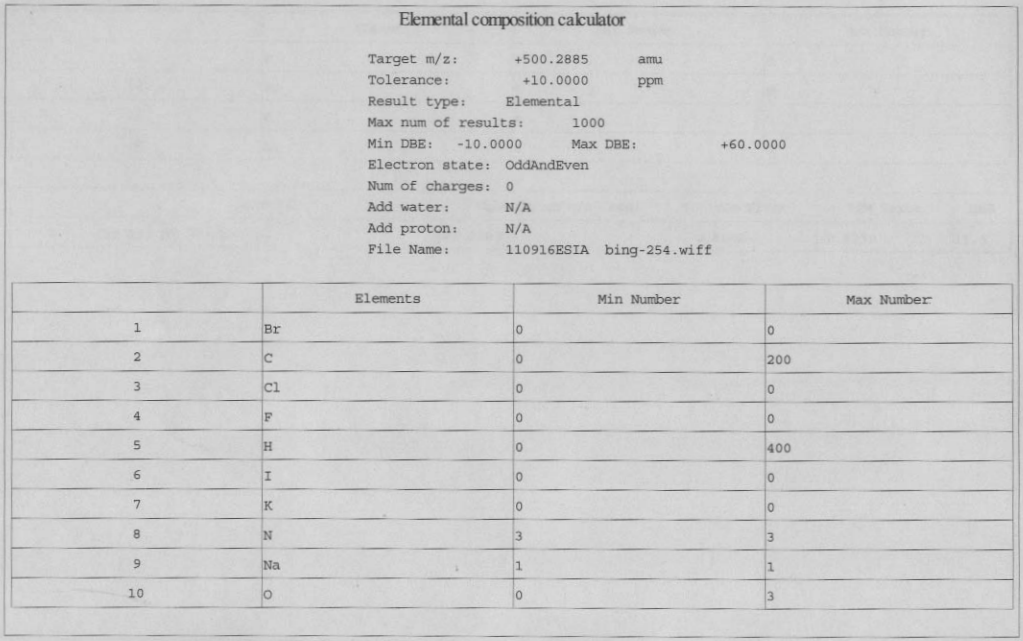


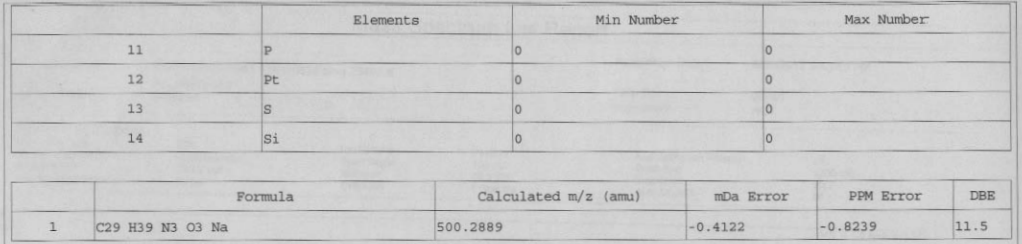


**Figure S14.** HR-ESI-MS spectrum of compound (**3**).


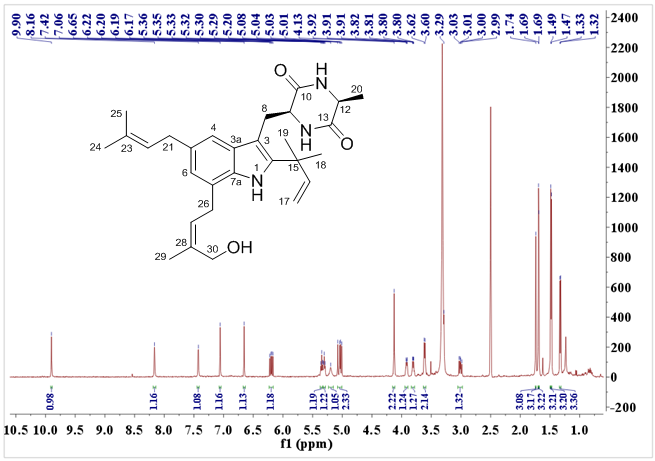


**Figure S15.** ^1^H-NMR (500 MHz, DMSO-*d*_6_) spectrum of compound (**3**).


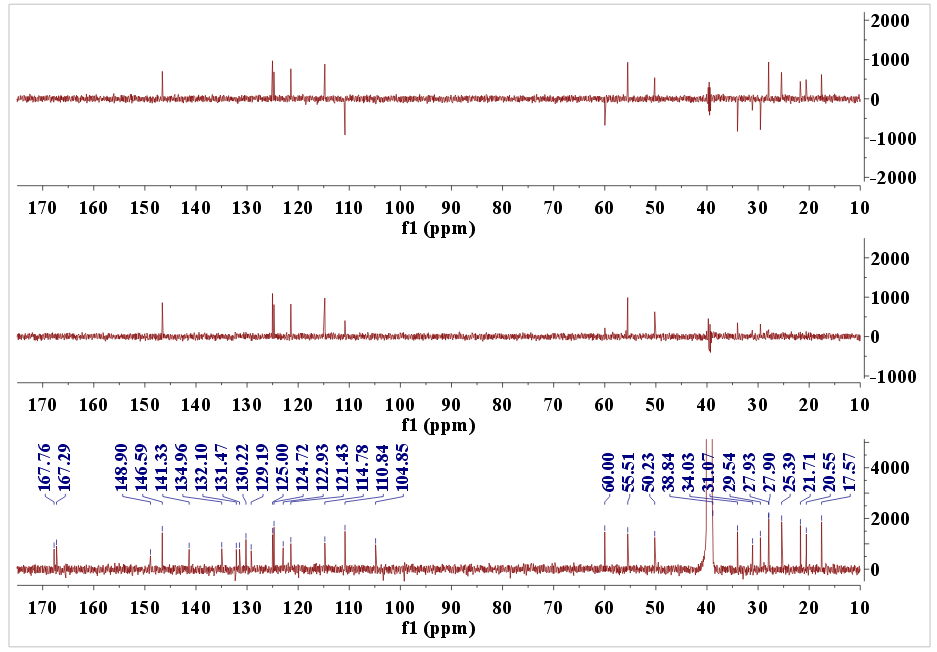


**Figure S16.** DEPT spectra of compound (**3**).


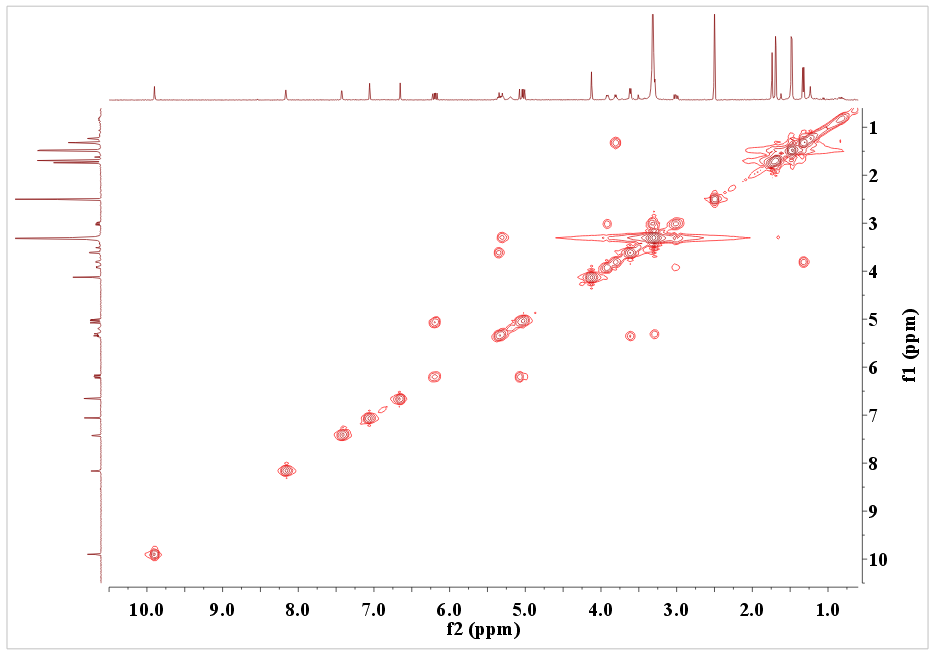


**Figure S17.** ^1^H–^1^H COSY spectrum of compound (**3**).


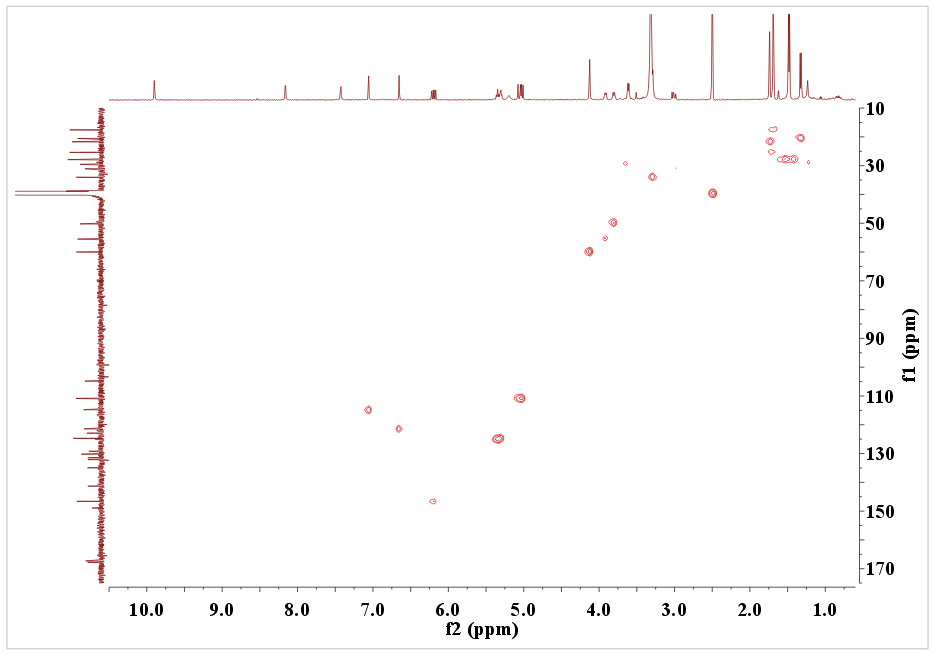


**Figure S18.** HSQC spectrum of compound (**3**).


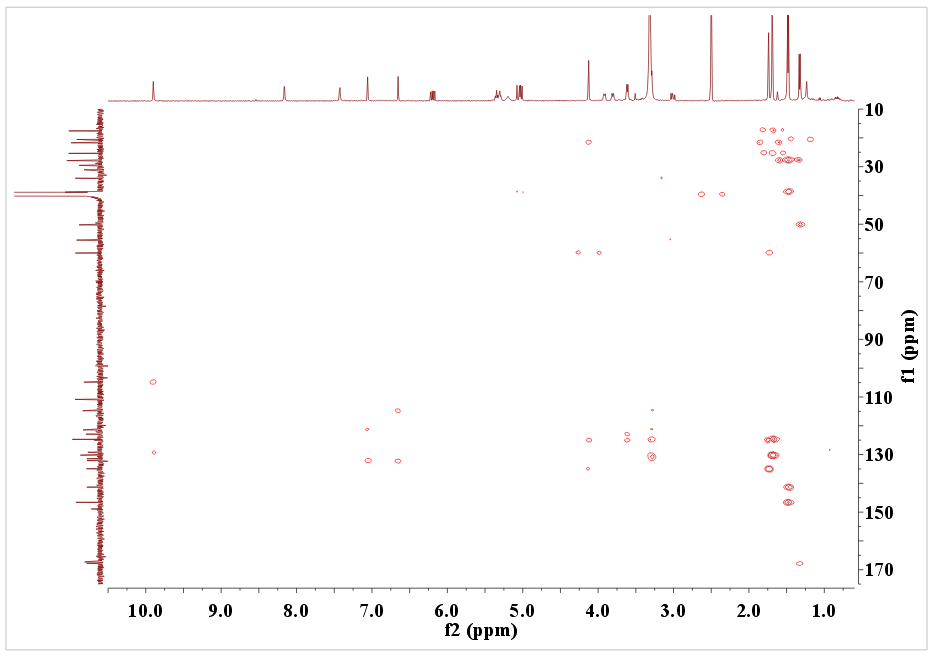


**Figure S19.** HMBC spectrum of compound (**3)**.


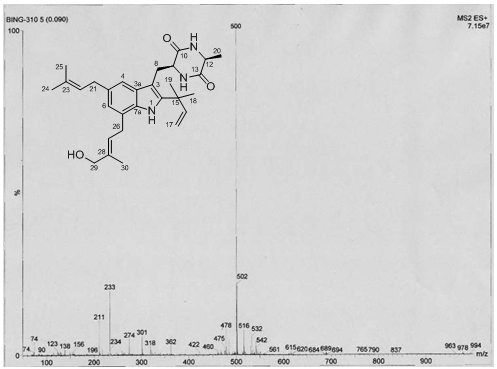


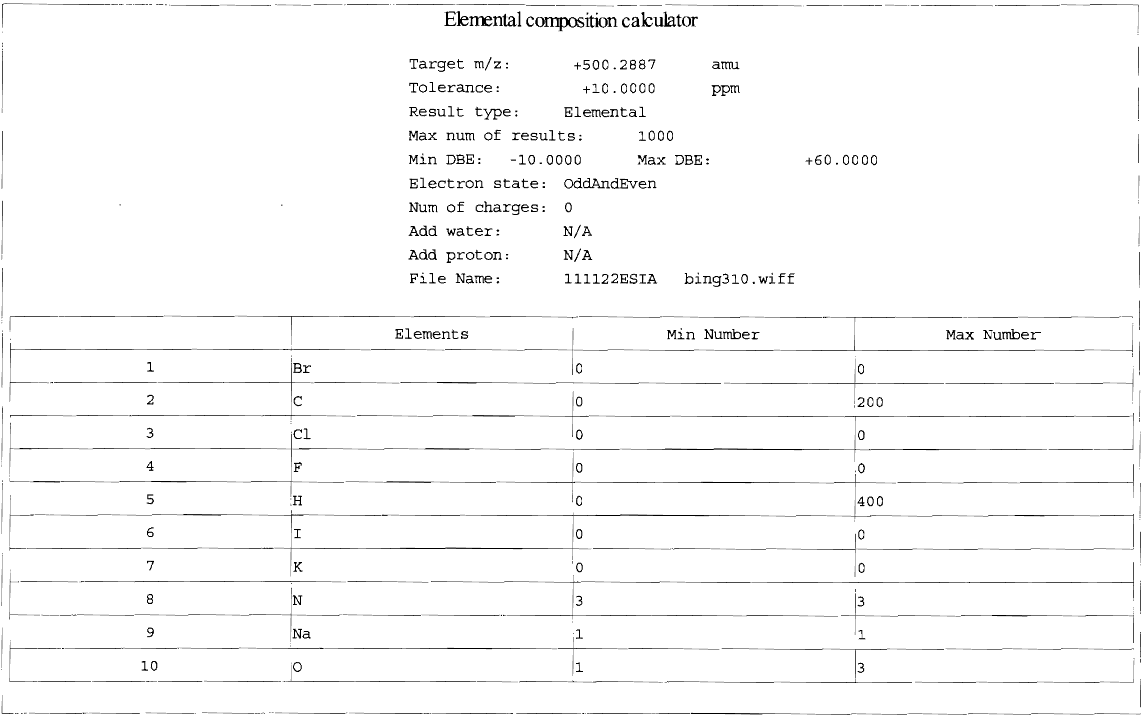


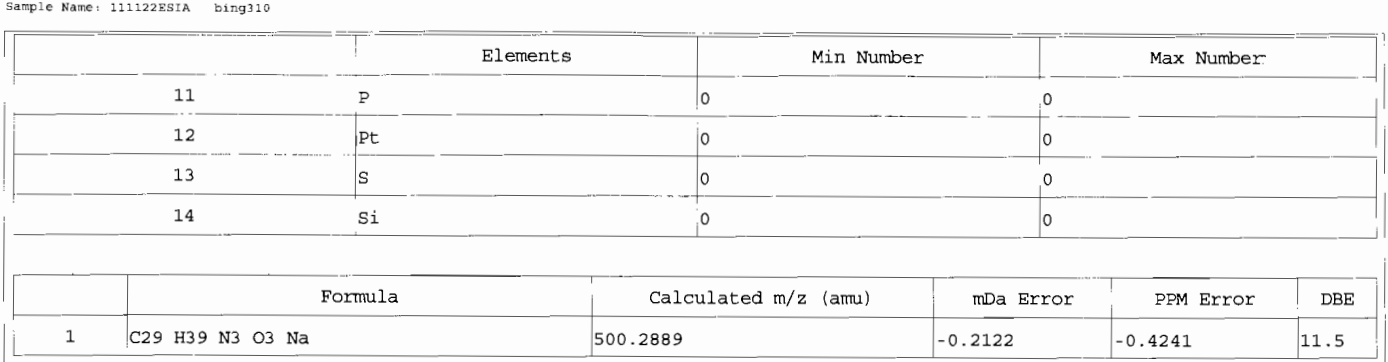


**Figure S20.** ESI-MS spectrum and HR-ESI-MS data of compound (**4**).


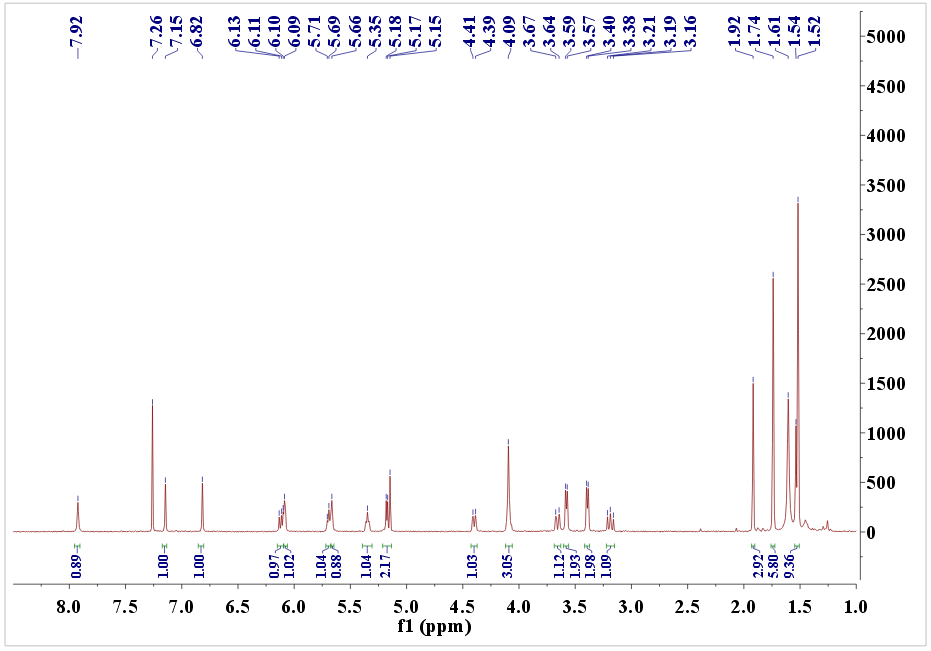

**Figure S21.** ^1^H-NMR (500 MHz, CDCl_3_) spectrum of compound (**4**).


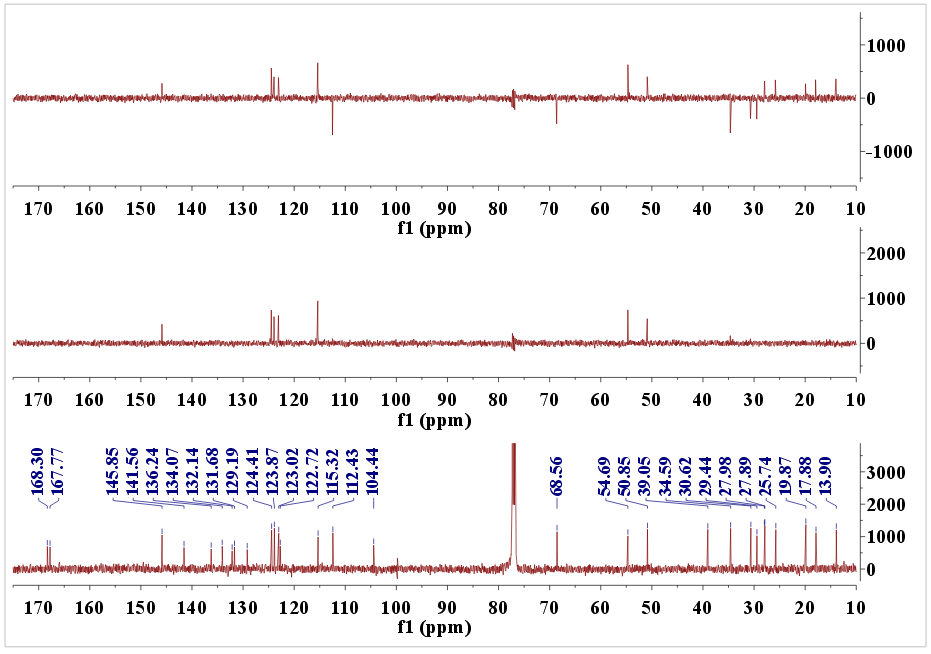


**Figure S22.** DEPT spectra of compound (**4**).


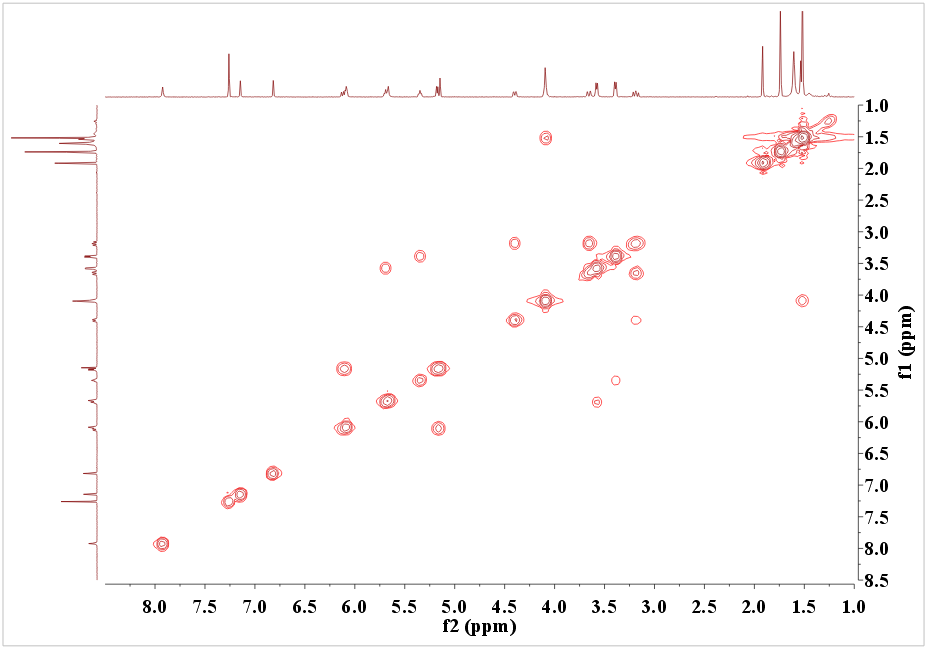


**Figure S23.** ^1^H–^1^H COSY spectrum of compound (**4**).


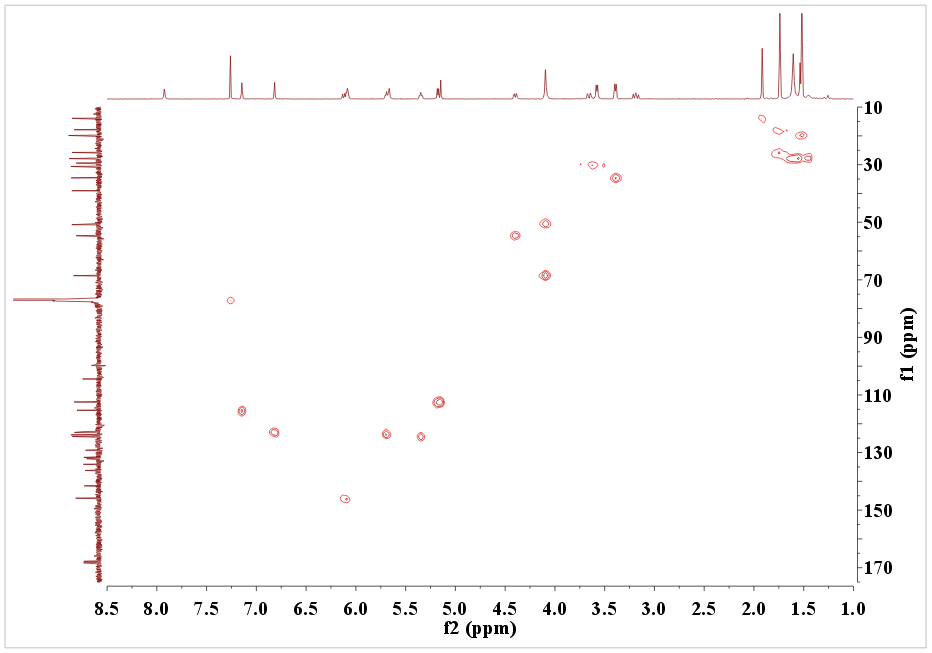


**Figure S24.** HSQC spectrum of compound (**4**).


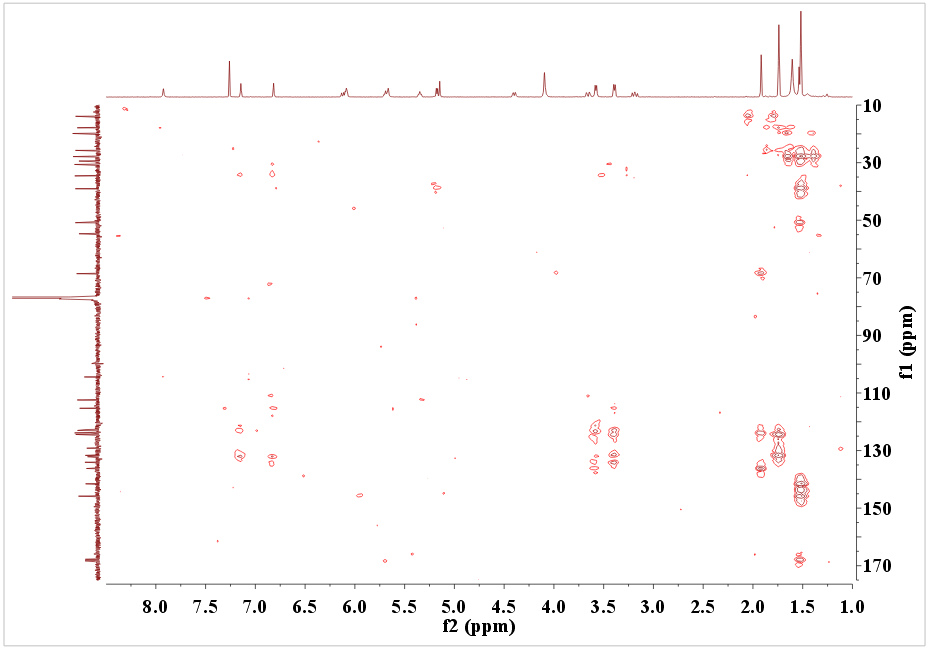


**Figure S25.** HMBC spectrum of compound (**4**).


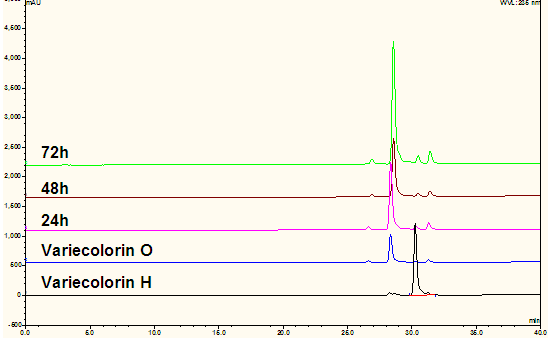


**Figure S26.** HPLC profiles of the mixture of variecolorin O and Si gel in solvent (CHCl_3_:MeOH = 1:1).
